# Characterization of research trends and prospects on hepatic echinococcosis over the past forty years: a bibliometric analysis

**DOI:** 10.1097/JS9.0000000000001319

**Published:** 2024-03-11

**Authors:** Tianen Li, Wei Su, Zhiqiang Wang, Xiao Wang, Xiaoguang Ma, Yigeng Cao, Rui Zhao

**Affiliations:** a Department of Hepatobiliary Surgery, Qilu Hospital of Shandong University, Jinan; b Department of Hepatobiliary Surgery, Qinghai Red Cross Hospital; c Department of Hematology, The Fifth People’s Hospital of Qinghai Province, Xining, Qinghai, People’s Republic of China

**Keywords:** bibliometric analysis, Bibliometrix, CiteSpace, hepatic echinococcosis, VOSviewer

## Abstract

**Background:**

The distribution of hepatic echinococcosis (HE) is extensive, significantly impacting public health and economic development. Therefore, analyzing global collaboration networks and tracking developmental trends over the past four decades are crucial. This study aimed to demonstrate collaboration in the field of HE and explore key topics and future directions.

**Materials and Methods:**

Bibliometric analyses were conducted using CiteSpace, Bibliometrix package of R, and VOSviewer software on HE-related studies from the Web of Science Core Collection published before 1 August 2023.

**Results:**

This study identified 2605 records published in 196 journals by 9860 authors from 2607 institutes in 90 countries. Publications significantly notably increased in 2021. Developing countries like Turkey and China made notable contributions, while developed countries like the USA had higher average citation rates. The largest nodes in every cluster of the collaboration network were Hacettepe University, Tehran University, Xinjiang Medical University, Salford University, and the University of Pavia, and the top-producing authors were Wen H, Vuitton DA, Gottstein B, and Craig PS. Keyword co-occurrence analysis suggested that surgical techniques and novel drugs targeting combined immune checkpoints are the main therapeutic approaches in the future.

**Conclusion:**

Although developing countries had significantly contributed to publications on HE, the citation rate for individual articles from developed countries was significantly higher. Additionally, advancements in surgical techniques and novel drugs targeting combined immune checkpoints may emerge as the next research focus and developmental direction.

## Introduction

Highlights

- This bibliometric analysis investigated hepatic echinococcosis over the past 40 years.
- Although hepatic echinococcosis was primarily documented in developing countries, developed countries significantly contributed to the citation rate of related individual articles.
- Advancements in surgical techniques and novel drugs targeting combined immune checkpoints may emerge as the next research focus and developmental direction.

Human echinococcosis is a parasitic disease caused by tapeworms of the genus *Echinococcus*, characterized by two primary forms: cystic echinococcosis (CE) and alveolar echinococcosis (AE)^1^. CE is globally distributed and has been reported in every continent except Antarctica. Conversely, AE is confined to the northern hemisphere, particularly in China and the Russian Federation. The WHO has estimated that echinococcosis accounts for 19 300 deaths and ~871 000 disability-adjusted life years worldwide annually, with hepatic echinococcosis (HE) accounting for 60–75% of these cases^2,3^.

In its early stages, HE is typically asymptomatic; however, as the cyst grows larger, it can cause significant mass effects on nearby structures, including the portal vein, hepatic vein, bile duct tree, right diaphragm, stomach, and kidney^4^. The space-occupying effect can lead to widespread complications in approximately one-third of patients, some of which may pose a threat to life, requiring urgent diagnosis and intervention^5^. The primary definitive approach for clinical diagnosis in patients with HE is the combination of surgical intervention and albendazole; however, owing to rapid disease progression, only a limited subset of patients can benefit from surgery^6,7^. The literature has reported that ex vivo liver resection and autotransplantation offer a chance for patients with end-stage HE; however, they also pose significant social and economic challenges^8,9^. Therefore, advancing early diagnosis and developing effective drugs and vaccines for controlling HE is significant. Such advancements will directly impact the future management of HE, which continues to be a global challenge.

Bibliometric analysis is a systematic approach that uses mathematical and statistical techniques to quantitatively evaluate a considerable number of studies and provide an invaluable tool for obtaining a comprehensive understanding regarding a specific research field. Additionally, it can provide researchers with a comprehensive literature review, help quantify and evaluate productivity in the field, and identify potential research directions^10,11^.

To the best of our knowledge, only two published papers have explored the trends of echinococcosis through bibliometric analysis^12,13^. Contrary to these two papers, this study exclusively focused on HE, employing more specific thematic terminology and a longer temporal scope. Consequently, it provides enhanced professional guidance regarding the current state and trend in echinococcosis research.

## Materials and methods

### Data source and search strategy

The Web of Science (WOS; data source: Science Citation Index Expanded) was chosen as the database for literature mining due to its extensive coverage of over 8000 high-quality journals from more than 100 countries worldwide, making it a comprehensive, systematic, and multidisciplinary authoritative database^14,15^. The WOS database retrieved a higher number of citations from articles, editorials, and letters, while Scopus database obtained a larger proportion of non-English and review citations^16,17^. The inclusion of studies funded by the industry, those investigating pharmaceuticals or medical devices, and those with collaborative authorship were all associated with a higher number of citations in Scopus and WOS compared to Google Scholar^18^. Therefore, selecting literature from WOS enables an analysis of the overall situation in HE research. Therefore, the WOS prioritizes the quality of its content coverage over quantity, making it a suitable choice for this bibliometric study.

A comprehensive online search was conducted in the field of HE using the Core Collection database WOS on 1 August 2023. The terms searched encompassed ‘hepatic/liver hydatid cyst*’ OR ‘hepatic/liver *Echinococcus granulosus*’ OR ‘hepatic/liver *Echinococcus multilocularis*’ OR ‘hepatic/liver echinococcosis’ OR ‘hepatic/liver Hydatidosis’ OR ‘hepatic/liver echinococcal disease’ OR ‘hepatic/liver cystic echinococcosis’ OR ‘hepatic/liver alveolar echinococcosis’. The publication time frame ranged from 1983 to 31 July 2023.

### Inclusion and exclusion criteria

The inclusion criteria encompassed published records exclusively written in English, with a specific focus on HE-related articles and reviews. To ensure consistent and accurate information collection based on a comprehensive analysis of published records, we excluded book chapter reviews, retracted publication articles, and cases of HE in nonhuman participants.

### Data processing

1. To mitigate any potential bias due to daily database updates, the search was conducted on a single day and was limited to studies published in the English language, including only original articles and reviews. In total, 143 items that met the inclusion criteria were retrieved.
2. Two groups of reviewers, who underwent standard selection training, independently screened the titles and abstracts. Full texts were retrieved as necessary. Any disagreements between the two groups were discussed and resolved.
3. Bradford’s law: Bradford’s law was used as a bibliometric indicator to evaluate the dissemination of scientific information^19,20^. In 1934, Bradford initially observed that when dividing all references in a specific subject into three groups or zones, the citations for the first zone would come from a concentrated ‘core’ group of journals^21,22^. The second zone would require more journals to achieve an equivalent number of citations, while the third zone would require exponentially more than the second. Moving from Zone 1 to Zone 3 involves a phenomenon known as ‘diminishing productivity’, described by Bradford and commonly referred to as Bradford’s law of scattering. This means that each zone contains a similar number of documents; however, the number of journals in which they are published progressively increases with each subsequent zone. As a result, this allows identification of the most widely used and highest impact journals in a given area of evaluation. The present study utilized core + zone 2 sources to mitigate the inclusion of predatory or low-quality journals that could potentially introduce bias into this analysis.

### Bibliometric analysis

All data in the WOS Core Collection were saved in plain text format, encompassing both full record and cited references. The bibliometric analysis was conducted using CiteSpace (version 6.2.R4), Bibliometrix package of R software (version 4.2.1), and VOSviewer (version 1.6.19).

The widely used statistical analysis tool, CiteSpace, developed by Chen in the Java environment, utilizes co-citation analysis theory and pathfinder network scaling to visually map literature in specific fields and reveal emerging trends in related disciplines^23,24^. CiteSpace can effectively facilitate researchers in gaining a comprehensive understanding of the research domains they are actively involved in: it not only provides an overview of the overall landscape within a specific field, but also emphasizes pivotal documents that play significant roles throughout the developmental trajectory^25,26^. In this study, the CiteSpace software was utilized to generate interactive visualizations that depict the structural and temporal patterns observed in developmental dynamics, future trends, hotspots, and key points.

The R language and environment was specifically designed for statistical computing and graphics, offering a high level of extensibility^27^. It enables automated analysis and the creation of new functions. Bibliometrix was an R package containing a range of functions designed for quantitative research in scientometrics^28–30^. In this study, it was utilized to: 1) provide an overview of the number of publications and citations in bibliometric analysis; 2) identify the annual cumulative occurrences of top keywords/terms; and 3) calculate the frequency of cooperation among countries.

VOSviewer was employed to construct and visualize author collaboration within a compilation of publications, as well as facilitate collaboration among countries, institutions, and high-frequency keywords^31,32^. Co-citation and co-occurrence analyses were conducted using VOSviewer in this study. Node size represented the number of publications, line thickness indicated the strength of relationships, and node colors denoted distinct clusters or periods^33^.

Analysis of the top 20 countries was conducted using SPSS17 (SPSS Inc.) via *t*-tests. A *P*-value of <0.05 was considered to indicate statistical significance.

## Results

### Search results

**Figure 1:**
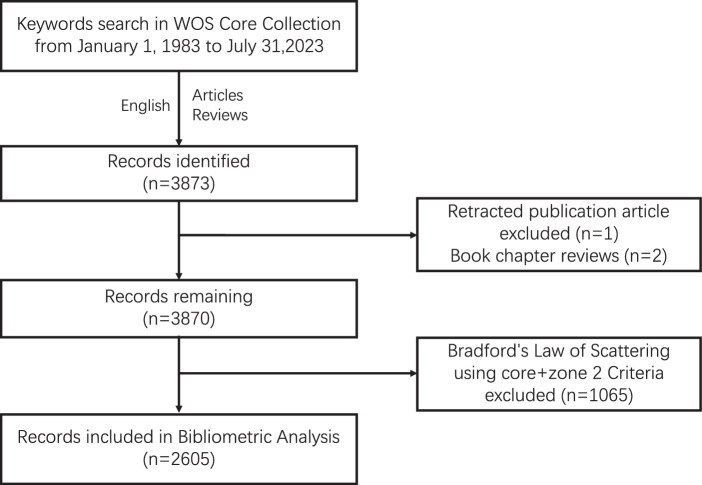
Identification and selection of records.

After applying the inclusion and exclusion criteria, a total of 3873 publications were identified from the WOS Core Collection. Two book chapter reviews and one retracted publication article were excluded after reviewing the titles and abstracts. Furthermore, the application of Bradford’s Law of Scattering to the cited journal list resulted in the exclusion of 1065 articles using core + zone 2 sources. Finally, a total of 2605 studies were included in the bibliometric analysis, with 3873 records identified as the number of publications (Fig. 1).

### Annual publication

**Figure 2:**
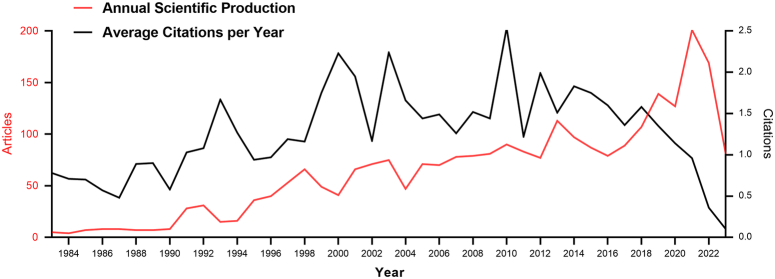
Trend of annual publication and citation numbers for hepatic echinococcosis from 1983 to 2023.

In the past four decades, academic journals have published a total of 2438 articles (93.59%) and 167 reviews (6.41%) on HE. The majority of publications comprised articles, effectively capturing the dynamic trends and advancements in the field of HE. The first report on the surgical management of HE was published in 1922, wherein a patient with HE was successfully treated by resecting the seventh and eighth ribs and subsequently inserting a drainage tube into the cavity^34^. The annual publishing volume has been steadily increasing since 1983, with a consistent annual growth rate of 7.21% (Fig. 2A). Additionally, before 1990, the number of annual publications was limited to only 4–8. However, a noticeable upward trend in the publication count was observed from 1991 to 2004, which became even more pronounced after 2005 (*n*=71), ultimately reaching a total of 201 publications in 2021. The average citation per year was also influenced by the annual number of published articles, and it reached its highest point in 2010 (Fig. 2B).

### Analysis of countries and regions

The global scientific community witnessed contributions from 90 countries in the field of HE. The findings showed that six countries have published over 100 records, including Turkey (531/2,605, 20.3%), China (305/2,605, 11.7%), Iran (173/2,605, 6.6%), India (141/2,605, 5.4%), Italy (114/2,605, 4.3%), and Germany (108/2,605, 4.1%). Although the number of records published by France, Switzerland, Greece, and the USA was below 100, no statistically significant differences were observed among the countries (*P*=0.16). Furthermore, their contribution was approximately equivalent to that of India, Italy, or Germany. Moreover, Italy (38.9 times) had the highest average number of citations per article, followed by France (34.1 times), Switzerland (30.4 times), the USA (28.5 times), Germany (28.2 times), and Turkey (18.7 times) (Table 1).

**Table 1:** Country production rank.

Rank	Country	Documents, *n* (%)	Citations, *n*	Average citations
1	Turkey	531 (20.3)	9904	18.7
2	China	305 (11.7)	2723	8.9
3	Iran	173 (6.6)	2359	13.6
4	India	141 (5.4)	1366	9.7
5	Italy	114 (4.3)	4430	38.9
6	Germany	108 (4.1)	3045	28.2
7	France	99 (3.8)	3377	34.1
8	Switzerland	84 (3.2)	2552	30.4
9	Greece	75 (2.8)	1699	22.4
10	USA	71 (2.7)	2023	28.5

Table 2 shows that there was no statistically significant difference the total number of published papers (*P*=0.16) and citations (*P*=0.98) between developed and developing countries. However, the average citation rate was significantly higher for developed countries than for developing countries (*P*=0.001).

**Table 2:** Analysis of the top 20 countries.

	Developed countries, *n*=11	Developing countries, *n*=9	*P*
Documents, *n*	800	1337	0.16
Citations, *n*	23 632	19 145	0.98
Average citations, *n*	28.9	14.5	0.001^*^

* *P*<0.05.

**Figure 3:**
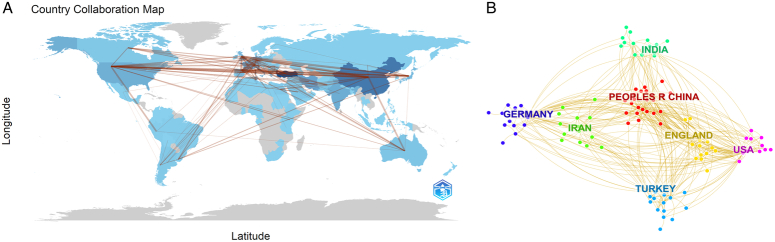
Distribution of countries and regions. Network visualization map of countries associated with hepatic echinococcosis created using Bibliometrix package of R software (A) and CiteSpace (B).

The top 50 levels of the most frequently cited or mentioned items were selected from each slice, thereby forming six clusters among 90 countries. A network map was subsequently generated (Fig. 3A). Active collaborations were noted among these countries, particularly between the USA, Turkey, Germany, and China (Fig. 3B).

### Analysis of universities and institutions

**Figure 4:**
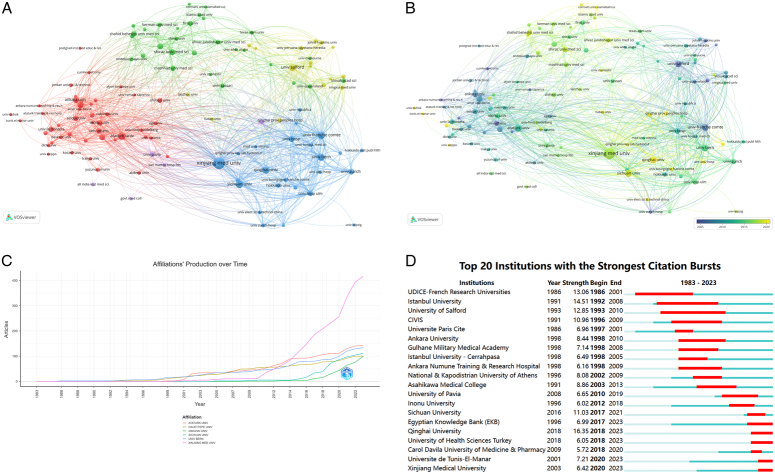
Distribution of universities and institutions. (A) Network map of universities and institutions (top 50). (B) Dynamics and trends of universities and institutions over time (top 50). (C) Productions over time in the top five universities. (D) Top 20 universities with the strongest citation bursts during 1983–2023.

The articles encompassed a total of 2607 institutions, with six universities contributing over 100 publications, including Xinjiang Medical University (417/2,605, 16.0%), Ataturk University (143/2,605, 5.4%), University of Bern (134/2,605, 5.1%), Sichuan University (112/2,605, 4.3%), Hacettepe University (100/2,605, 3.8%), and Qinghai University (100/2,605, 3.8%) (Table 3). To date, institutions within the same countries exhibit strong collaboration, whereas international exchanges and cooperation remain relatively limited (Fig. 4A).

**Table 3:** Institution production rank.

Rank	Institution	Articles
1	Xinjiang Medical University	417
2	Ataturk University	143
3	University of Bern	134
4	Sichuan University	112
5	Hacettepe University	100
6	Qinghai University	100
7	University of Franche-Comte	92
8	University of Zurich	91
9	Tehran University of Medical Sciences	89
10	University of Ulm	85

Cooperation network analysis was conducted among universities or institutions, which yielded five clusters on a network map and an overlay visualization featuring the top 50 frequencies. Hacettepe University (*n*=44, Cluster 1), University of Tehran (*n*=29, Cluster 2), Xinjiang Medical University (*n*=24, Cluster 3), Salford University (*n*=16,Cluster 4), and the University of Pavia (*n*=5, Cluster 5) were the largest nodes in each of the five clusters. Cluster 1 was the largest, involving 44 nodes representing various universities or institutions, whereas Cluster 5 was the smallest with only five nodes (Fig. 4A, B). Furthermore, the findings indicate that before 2010, Xinjiang Medical University, University of Bern, Sichuan University, and Ataturk University were among the pioneering institutions in primary and fundamental HE research (Fig. 4C). In addition, these studies have gradually gained increasing citations among various universities and institutes, particularly at Xinjiang Medical University and Qinghai University since 2017 (Fig. 4D).

### Analysis of authors and journals

**Figure 5:**
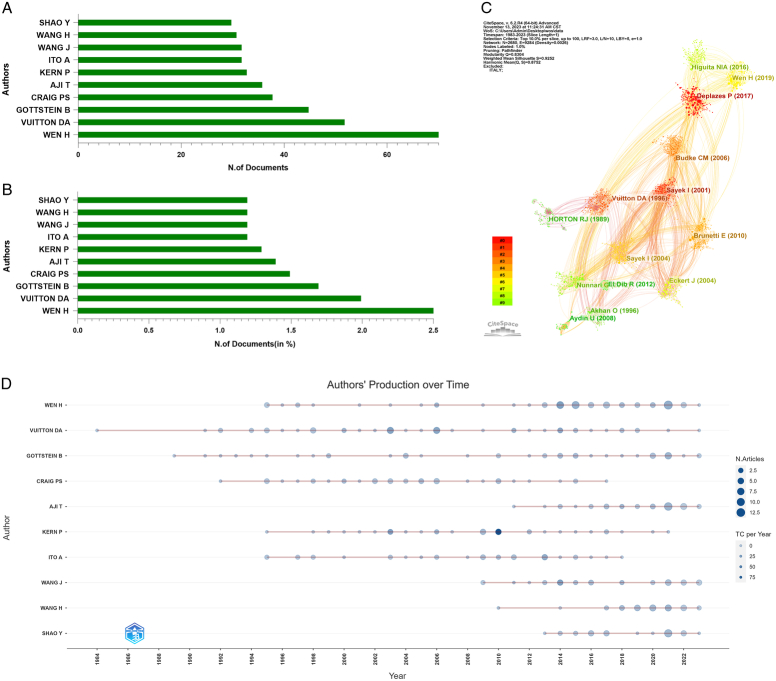
Distribution of authors. (A) Number of documents in the top 10 authors. (B) Number (in %) of documents in the top 10 authors. (C) Network map of authors (top 50). (D) Dynamics and trends of authors over time (top 10).

Overall, 9860 authors contributed to HE-related publications. Wen H (72/2605, 2.7%), Vuitton DA (52/2605, 2.0%), Gottstein B (45/2605, 1.7%), Craig PS (38/2605, 1.4%), and Aji T (36/2605, 1.3%) were the top five authors (Fig. 5A, B). The network map and overlay visualization of the top 50 authors who demonstrated high cooperation levels in their productivity formed distinct clusters (Fig. 5C). Furthermore, the findings indicated an increasing trend in HE research among various universities and institutes since 2014, as evidenced by an increasing number of publications from the top 10 authors (Fig. 5D).

A total of 196 journals published articles pertaining to HE between 1 January 1983 and 31 July 2023. The findings showed that the most productive journal was *Acta Tropica* (86/2605, accounting for 3.3% of all publications), with the highest number of total citations (3662 times). Meanwhile, *World Journal of Surgery* had an h-index score of 33 and accounted for 2.4% (65/2605) of all publications.

Of the total of 2605 papers, the top 10 publications, ranked by citation, are listed in Table 4. The first paper, published in 2010 by Brunetti *et al*.^35^ in *Acta Tropica*, presented an expert consensus on the diagnosis and treatment of CE and AE in humans. It garnered significant attention with 1129 citations, surpassing the second paper (503 citations)^36^. The 10 records comprised four publications in *RadioGraphics*, covering topics such as radiologic and pathologic features^37^, differential computed tomography (CT) and MRI features in HE^45^, and radiologic–pathologic correlation in the infected liver^46^. The latest paper^46^, which was published in 2004, was cited 198 times.

**Table 4:** The most local cited references.

Rank	Paper	DOI	Total citations	TC per Year
1	Brunetti E, 2010, Acta Tropica^35^	10.1016/j.actatropica.2009.11.001	1129	80.64
2	PEDROSA I, 2000, RadioGraphics^36^	10.1148/radiographics.20.3.g00ma06795	503	20.96
3	POLAT P, 2003, RadioGraphics^37^	10.1148/rg.232025704	325	15.48
4	TORGERSON PR, 2010, Plos Neglect Trop Dis^38^	10.1371/journal.pntd.0000722	311	22.21
5	KERN P, 2003, Merg Infect Dis^39^	10.3201/eid0903.020341	277	13.19
6	Mortele KJ, 2001, RadioGraphics^40^	10.1148/radiographics.21.4.g01jl16895	266	11.57
7	Junghanss T, 2008, Am J Trop Med Hyg^41^	10.4269/ajtmh.2008.79.301	214	13.38
8	Kern P, 2006, Parasitol Int-a^42^	10.1016/j.parint.2005.11.041	208	11.56
9	Horton Rj, 1997, Acta Tropica^43^	10.1016/S0001-706X(96)00640-7	201	7.44
10	Mortele KJ, 2004, RadioGraphics^44^	10.1148/rg.244035719	198	9.9

### Analysis of co-citation reference

**Figure 6:**
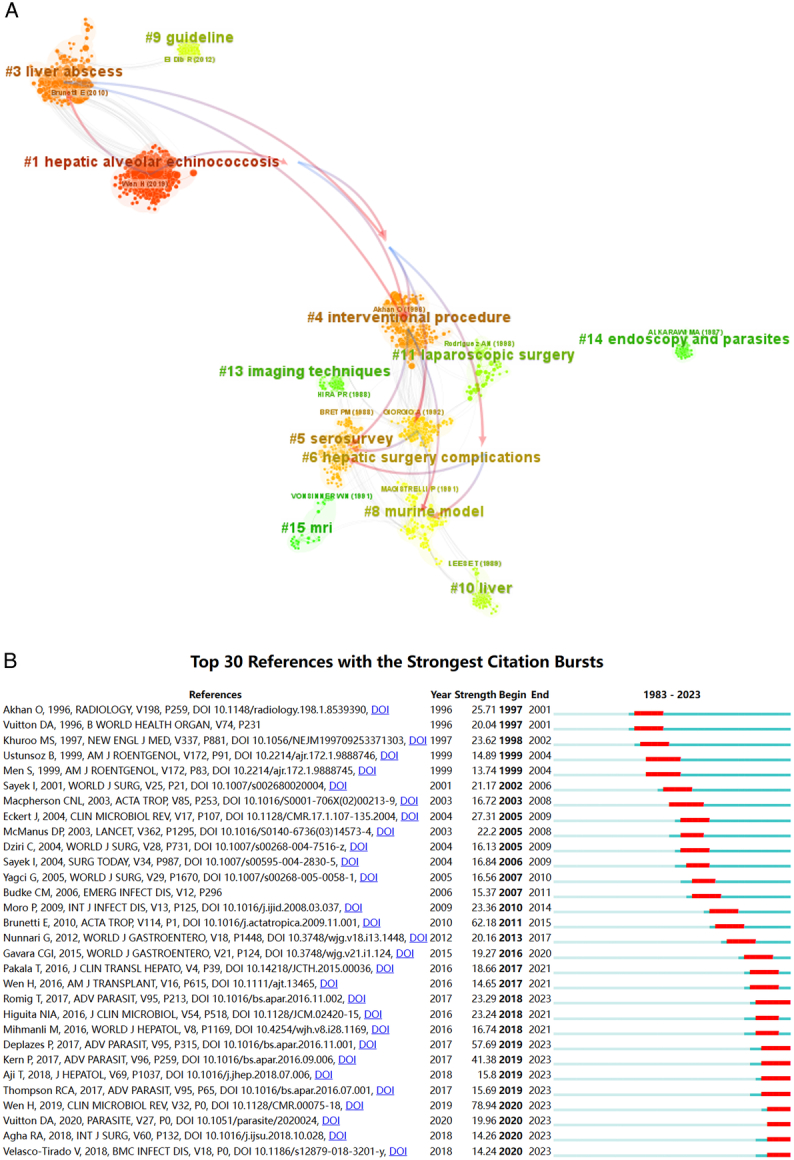
Distribution of co-citation references. (A) Co-citation network of references. (B) Top 30 references with the strongest citation bursts during 1983–2023.

CiteSpace software was employed to visually depict the co-citation network of references, which were classified into 12 distinct co-citation clusters, including ‘hepatic alveolar echinococcosis’ (Cluster 1, *n*=233), ‘liver abscess’ (Cluster 3, n=200), ‘interventional procedure’ (Cluster 4, n=182), ‘serosurvey’ (Cluster 5, n=126), ‘hepatic surgery complications’ (Cluster 6, n=107), ‘murine model’ (Cluster 8, n=88), ‘guideline’ (Cluster 9, n=86), ‘liver’ (Cluster 10, n=64), ‘laparoscopic surgery’ (Cluster 11, n=46), ‘imaging techniques’ (Cluster 13, n=39), ‘endoscopy and parasites’ (Cluster 14, n=29), and ‘MRI’ (Cluster 15, n=26) (Fig. 6A).

The top 30 references exhibiting the most significant citation bursts are depicted in Figure 6B. The initial occurrence of a citation burst occurred in 1997, whereas the majority of bursts were observed between 2010 and 2020, encompassing 17 citation peaks within this decade, in contrast to only 13 peaks recorded over the past 13 years.

### Analysis of keywords

**Figure 7:**
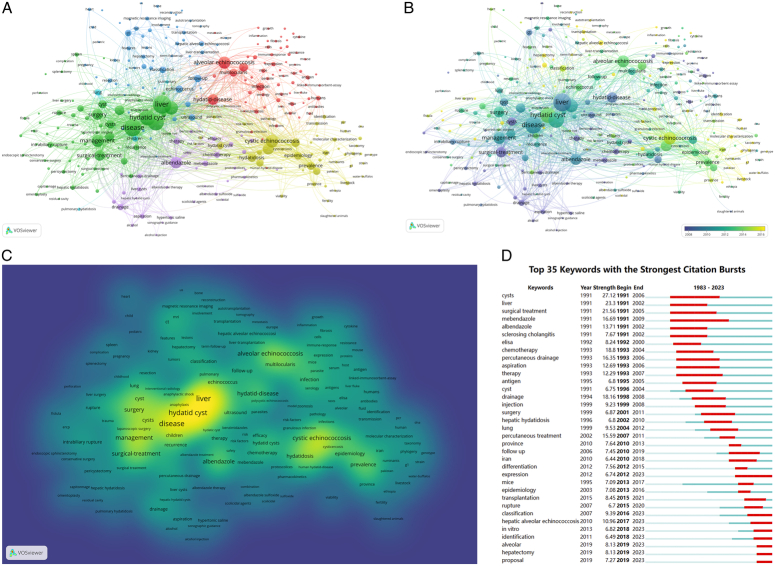
Distribution of keywords. (A) Network map of the top 10% frequency keywords. (B) Dynamics and trends of the frequency keywords over time (top 10%). (C) The most common keywords in hepatic echinococcosis research. The brightness of each node represents the frequency of keywords. (D) Top 35 keywords with the strongest citation bursts during 1983–2023.

The top 10% of the most frequently cited or mentioned items were selected from the 2605 published records, resulting in 322 extracted keywords and the formation of six clusters in the network map (Fig. 7A). ‘Liver’, ‘disease’, and ‘echinococcosis’ were the three largest nodes in the different clusters.

Cluster 6 is characterized by the node ‘children’ and includes topics related to childhood, lung, treatment options, and simultaneous surgical procedures. Cluster 3 showcases advancements in HE management, featuring novel diagnostic techniques and invasive surgical interventions like biliary puncture, drainage, and endoscopic retrograde cholangiopancreatography, along with associated complications such as bile leakage. Cluster 5 highlights key aspects of surgical intervention for HE, encompassing significant keywords like albendazole, percutaneous treatment, drainage, alcohol injection, and laparoscopic procedures. With 75 keywords, Cluster 2 serves as the central hub of HE studies. Notably, AE emerges as a prominent node in Cluster 2, showing strong associations with various keywords including Echinococcus granulosus, infection, expression, antibody, protein, and *in vivo/vitro* studies, indicating its significant focus area. Additionally, these findings underscore the continued importance of AE-related topics. Cluster 4 primarily focuses on the epidemiology of HE, featuring 65 items with a main emphasis on CE, prevalence rates, and epidemiological analyses. Cluster 1 encompasses advancements in hepatectomy for HE treatment, incorporating diagnostic criteria from CT and MRI, computer-assisted three-dimensional liver imaging, conventional partial liver resection, laparoscopic hepatectomy, ex vivo liver resection, and autotransplantation. Together, these developments highlight the comprehensive application of hepatectomy technology in HE treatment.

The overlay visualization of keywords between 2005 and 2020 is illustrated in Figure 7B and C. The largest nodes such as ‘liver’, ‘disease’, ‘management’, and ‘surgical treatment’ were identified before 2010. Subsequently, the frequency of several keywords such as ‘laparoscopic hepatectomy’, ‘autotransplantation’, ‘MRI findings’, ‘PCR’, ‘gene expression’, ‘mechanisms’, and ‘in vivo/vitro’ exhibited a gradual and substantial increase, capturing significant attention from 2015 to 2020.

The data showed that the top 35 keywords exhibiting citation bursts started in 1991 (Fig. 7D). Similarly, from 1991 to 2006, the ‘cysts’ stood out as the primary research focus. Moreover, at the onset of 2012, several computer algorithm-related keywords such as ‘classification’, ‘expression’, ‘in vitro’, and ‘hepatectomy’ demonstrated the most prominent citation bursts.

## Discussion

Herein, a comprehensive overview of global research collaborations, recent advancements, and emerging trends in the field of HE is provided. In particular, bibliometric analysis enables the visualization of the current hotspots and trends in this field based on published records^10,11^. We analyzed the most productive authors and institutions by journal and co-citation references, illustrating research groups with collaboration networks over the past four decades. The period from 1 January 1983 to 31 July 2023 witnessed the publication of a total of 2605 records in 196 journals originating from 90 countries and affiliated with 2607 institutions. The country with the highest absolute productivity was Turkey, followed by China, Iran, and India (all Asian countries). This was further followed by Italy, Germany, and other European countries, including France, Switzerland, and Greece. The findings indicated a significant disparity in the prevalence of articles regarding HE between developing and developed countries, with a notably higher occurrence observed in developing countries. The high incidence of HE in developing countries could be attributed to the prevalence of wild dogs as definitive hosts and unsanitary hygiene practices, which pose significant challenges as a global public health concern, as reported by numerous studies^47–49^. Moreover, Asian countries published a considerably greater number of articles than European nations, highlighting an inverse correlation with per capita gross domestic product. However, in terms of the number of publications on HE reported since 2010, China surpassed Turkey, securing the leading position. This achievement may be attributed to the Chinese government’s implementation of the ‘Hepatic Echinococcosis Elimination’ campaign in 2010 and increased investment in research on HE. Nevertheless, this trend is also related to other factors, including advancements in diagnostic techniques, evolving research priorities, and funding allocations.

In 1761, the first autopsy of a patient diagnosed with echinococcosis was documented in Iceland. Furthermore, the first documented case of surgical management of HE was reported in 1922, wherein a patient with HE was successfully treated by resecting the seventh and eighth ribs and inserting a drainage tube into the cavity^34^. The attention obtained from governments worldwide resulted in a 7.21% increase in the publication of articles on HE since 1983, with a notable upsurge in publications observed after 2016. A significant increase in the number of published papers and rate of citation among Chinese universities (Xinjiang Medical University, Sichuan University, and Qinghai University) during this period may have played a pivotal role in driving the surge of global articles after 2016. Additionally, several studies representing fundamental milestones in HE development were noted. For example, the use of albendazole in human CE treatment demonstrated a significant advancement over the course of 12 years of experience^43^. Subsequently, four seminal papers were published in *RadioGraphics* between 2000 and 2004, encompassing radiologic–pathologic features and differential CT and MRI characteristics of HE, along with radiologic–pathologic correlation in the infected liver^36–39,45,46^. Furthermore, in 2010, Brunetti E *et al*.^35^ published an expert consensus on the diagnosis and treatment of CE and AE in humans in *Acta Tropica*. Since 2011, the articles that have been widely cited predominantly focused on retrospective studies or advancements in imaging techniques; however, significant strides have also been made in the realm of surgical methodologies and molecular experimental investigations. Aji *et al*.^8^ reported the successful implementation of ex vivo liver resection and autotransplantation as a viable alternative to allotransplantation in a large cohort of patients with end-stage hepatic AE. In the field of molecular experimentation, enhancing the efficacy of albendazole is a focal point in current research. For instance, albendazole has been used in combination with novel materials and formulated into albendazole–chitosan microspheres^50^ as well as utilized alongside novel immune checkpoints to exert its effects^51^.

Turkey (531/2,605, 20.3%) published the highest number of articles worldwide, whereas the highest average citation rate was observed in Andorra (124 times). In contrast to other countries, the high genetic diversity and abundance of intermediate hosts for *Echinococcus granulosus* in Turkey, combined with the close contact between local residents and dogs due to their living habits, contributed significantly to the high occurrence of HE in Turkey^52–54^. Although Andorra and Thailand (39 times) ranked first and second in terms of average citation rate, respectively, with only one published article each, we identified Italy (38.5 times) to be more compelling in terms of the impact of a single article on HE. This is primarily because the first report of HE was reported by esteemed experts in Italy, with a citation rate exceeding twice that of the second-ranked article. The numbers of citations and documents varied, as did the collaboration level among countries and regions. Notably, the USA, China, Germany, and Turkey exhibited robust cooperation with higher total link strengths than other countries. We believe that publications from Italy had a deeper impact on HE, although the number of publications by relevant authors from Italy was significantly less compared with that from Turkey and China. Furthermore, we observed that Xinjiang Medical University in China had one of the highest numbers of publications in this field, indicating an epidemic trend of HE. Among the 2607 universities or institutions worldwide, European universities (University of Bern, Ulm University, University of Zurich, and University of France-Comte), Chinese institutes (Xinjiang Medical University, Sichuan University, and Qinghai University), Turkish universities (Ataturk University and Hacettepe University), and the Tehran University of Med Sci in Iran had taken leading places in HE development with steady collaborations in global groups. Moreover, among the 9860 authors who contributed to the publications, the top 50 belonged to collaborative networks mainly involved in research from the mentioned countries and institutes. Interestingly, although China holds the second position in terms of number of published articles, it ranked last in terms of the average citation rate. Upon analysis, we found two aspects that may contribute to this phenomenon: (1) a low average citation rate due to a large volume of papers and (2) insufficient impact of the articles. Multicenter, in-depth studies often integrate resources and methodologies, rendering their research outcomes and articles more impactful^55^. Conversely, some single-center studies, despite possessing ample case resources, lack research experience and employ ineffective methodologies, yielding a plethora of low-quality and low-impact studies^56^. This underscores the critical importance of deep collaboration among diverse countries and research institutions. The sharing of case resources, experiences, and research methodologies among various research institutions, or even across different countries, can enhance the depth and breadth of research, amplify the impact of research outcomes and articles, and yield studies with greater clinical translational value for the diagnosis and management of HE.

HE is often expensive and complex to manage, frequently necessitating extensive surgical intervention and/or prolonged pharmacotherapy. Therefore, to understand the epidemiological status of the disease and implement preventive measures in high-risk areas as well as establish priorities, using statistical data is crucial. The analysis of 196 journals revealed that a subset of 20 journals made the most significant contributions. Remarkably, *Acta Tropica* emerged as the leading journal with an impressive publication count of 86 papers and the highest number of citations, with a total of 3662. Additionally, *World Journal of Surgery* achieved distinction by attaining a remarkable h-index score of 33. Moreover, all the top-10 most-cited papers were published in prestigious journals, and these records focused on human HE diagnosis and treatment as well as advancements in imaging techniques^36–39,45,46^. Collectively, these journals have received >300 citations within the past decade. In the co-citation analysis, knowledge on ‘hepatic alveolar echinococcosis’, ‘liver abscess’, ‘hepatic surgery complications’, ‘murine model’, ‘guideline’, and ‘laparoscopic surgery’ were the basis of HE research, and most references with citation bursts appeared between 2010 and 2020.

The visualization map of keywords consisted of six clusters, which served as a means for investigating crucial topics and potential future directions. Cluster 1, characterized by the highest number of keywords (*n*=77), encompassed the prevalence and epidemiological analysis of HE. Cluster 2 represented the advancements made in the understanding of infection, transmission, and prevention of AE. Clusters 3, 4, and 5 encompassed the advancements in hepatectomy, management, and surgical intervention for HE. Cluster 6 mainly referred to the developmental process of children with echinococcosis. The overlay visualization of keywords between 2005 and 2020 showed a shift in the frequency of certain terms. Before 2010, ‘disease’ and ‘management’ were the prominent terms whereas after 2012, the focus shifted toward ‘laparoscopic hepatectomy’, ‘autotransplantation’, and ‘gene expression’.

The shift in keywords indicated a transition in the approach for treating HE, moving from a focus on postsymptom improvement toward an increasingly preventive and control-oriented strategy driven by the research and development of new drugs and the application of innovative technologies. The main reasons for this transformation include the extensive utilization of minimally invasive techniques such as laparoscopy, further advancement of molecular diagnostic techniques such as RNA sequencing, and continued development of new drug-loaded materials. Additionally, a gradual increase in attention from government agencies toward HE was observed. Qiu *et al*.^57^ and Aji *et al*.^8^ conducted ex vivo liver resection and autotransplantation in 228 and 69 cases, respectively, yielding favorable outcomes, thereby providing a novel therapeutic approach for patients afflicted with end-stage HE. The findings of a retrospective study comparing laparoscopic and open treatment for HE suggested that laparoscopic hepatectomy is a feasible and secure approach for patients with stage 1 AE, without any impact on early disease recurrence^58^. Through RNA sequencing technology, a previous study identified a multitude of differentially expressed genes in the peripheral blood of patients afflicted with HE, thereby presenting novel targets for pharmacological intervention^59^. The combination of albendazole with novel materials (such as solid dispersion^60^, liposomes^61^, nanoparticles^62,63^, microspheres^50^, and self-microemulsion^64,65^) has been reported to enhance hepatic drug concentrations, thereby facilitating disease control; however, certain limitations still exist^66^. Moreover, during investigation on the correlation between HE and immune sites, we notably revealed that the downregulation of PD-1/PD-L1 in the liver can effectively impede the pathophysiological progression caused by parasites through modulation of natural killer T (NKT) cells and regulatory T cells (Tregs), as evidenced by in vivo animal studies^67,68^. This presented a novel perspective for the treatment of HE. The publication of these articles further substantiated the shift in keywords, additionally suggesting that the advancement of surgical techniques and development of novel drugs targeting combined immune checkpoints are likely to emerge as the predominant therapeutic approaches in the future.

Bibliometric analysis provides researchers with insights into the trends of knowledge within specific fields, serving as a key reference for exploring the intrinsic connections among scientific knowledge. However, this study had several limitations. Firstly, our search was initially limited to the WOS Core Collection and only included English-language publications, which may have introduced selection biases. The exclusion of Chinese publications might have led to an underestimation of contributions from Chinese researchers. Secondly, relying on software tools such as CiteSpace, the Bibliometrix package in R software, and VOSviewer may have inadvertently overlooked certain information, despite producing similar results. Thirdly, bibliometric analysis itself has limitations, including: (1) Its primary method involves collecting, screening, and organizing relevant publications, offering an overview of literature but lacking depth for detailed analysis. (2) Citation data can be influenced by factors such as self-citation, language bias, and disciplinary citation practices, impacting the reliability and validity of bibliometric analysis. (3) Journal impact factors and other metrics at the journal level have faced criticism for potentially misrepresenting the true impact of a study when evaluating individual researchers or articles. Despite these limitations, the bibliometric analysis conducted on HE-related studies provided valuable insights into the complex interactions among authors, institutions, and countries, shedding light on emerging trends and future directions in this field.

## Conclusions

Since 1983, the number of publications on HE has considerably increased. Turkey demonstrated the highest number of publications worldwide, followed by China, Germany, and the USA. Correspondingly, collaboration networks primarily comprised institutes and authors from these four countries. Moreover, Xinjiang Medical University from China demonstrated the highest number of publications, with *Acta Tropica* emerging as the leading publication journal in this field, boasting an impressive count of publications and citations. The focus has shifted from ‘disease’ and ‘management’ to ‘laparoscopic hepatectomy’, ‘autotransplantation’, and ‘gene expression’ in the context of HE. This suggests that advancements in surgical techniques and development of novel drugs targeting combined immune checkpoints are likely to emerge as predominant therapeutic approaches in the future.

## Ethical approval

The study was actually just a Bibliometric Analysis, not something that involved actual patients.

Ethical approval is not applicable.

## Consent

The article has already covered the above.

## Sources of funding

All sources of funding have been declared at the end of the text.

## Author contribution

T.L., W.S., Z.W.: study design, data analysis, and manuscript writing; X.W., X.M., Y.C.: study design and data collection; R.Z.: supervision. All authors contributed and reviewed the manuscript.

## Conflicts of interest disclosure

The authors have declared that no competing interests exist.

## Research registration unique identifying number (UIN)

1. Name of the registry: not applicable.
2. Unique identifying number or registration ID: not applicable.
3. Hyperlink to your specific registration (must be publicly accessible and will be checked): not applicable.

## Guarantor

Rui Zhao.

## Data availability statement

All data and related metadata underlying the findings reported in submitted manuscript was conducted using the Core Collection database Web of Science (WOS) (data source: Science Citation Index Expanded).

## Provenance and peer review

My paper was not invited.
